# The Value of Proximal Contact*Extracted from “Around the Table” in the *Dental Items of Interest* for May, 1918.

**Published:** 1918-06

**Authors:** F. H. Orten


### THE VALUE OF PROXIMAL CONTACT*

BY F. H. ORTEN, D.D.S.

Teeth serve to render each other mutual support, so that when a tooth is lost from the arch the approximating teeth tend to work their way into the space left vacant, thus acquiring an abnormal range of movement. Now, in these cases, it has been repeatedly demonstrated that the use of fixed bridgework in order to immobilize these teeth to a certain extent, has served to prolong their life and usefulness for many years.

* Extracted from "Around the Table" in the *Dental Items of Interest* for May, 1918.

Assuming for example the simple case of the loss of the first lower molar. There invariably occurs as a consequence, a gradual mesial-lingual tipping of the second and third molars, and a buccal tipping of the opposing teeth. The change is gradual, extending over a period of years. It may be very much slower than the changes forced by orthodontic appliances. In the early stages such a condition presents no indications of pathology, no signs of pyorrhea; and the teeth are not loose, as that term is ordinarily understood. The teeth simply do not have the same support that they formerly had and still need.

One of the simplest methods at our command for the correction of this condition, as well as for the restoration of normal occlusion and articulation, a method which prevents further changes in the arches, in my opinion, is the use of a properly constructed fixed bridge. The comparison with a surgeon's splint is wholly inapplicable, and the two cases are not at all parallel. A surgeon's splint is used to immobilize a tissue temporarily, so as to make more favorable the conditions of repair; a fixed bridge is intended to act as a substitute for lost tissue, and to restore lost function. A properly constructed bridge would not absolutely immobilize the teeth, though it would prevent the teeth used as piers from moving except in unison. My experience is that the majority of fixed bridges have altogether too much, instead of too little, movement. Fixed bridgework as such should not be made responsible for what is merely an ordinary failure to pay attention to certain essential requirements of good dentistry.

I would not have it understood that I am advocating fixed bridgework in general, as opposed to removable bridgework, in general. I am, on the contrary, thoroughly distrustful of all panaceas, by whatever name they are called. I am convinced that fixed bridgework has a limited range of application. Merely to illustrate my position, I will say that I am among the pioneers in the construction of removable bridgework. I have one patient for whom I constructed a removable bridge eighteen years ago, since several times repaired, and still doing good service. I also have several patients for whom I constructed fixed bridgework in 1898, and two in 1899. Within the last two years I have inspected all these cases, and have found the abutment teeth still firm—I mean that they are as firm as the corresponding teeth on the opposite side of the arch, which have not been immobilized. Considering that I was at that time quite ignorant of essential requirements, and that my experience was limited, the fact that good results were obtained seems to me sufficient justification of my insistence that further proof ought to be presented before the profession can justly be called upon to abandon this apparently valuable method. Nor is my experience in this respect exceptional. It is difficult not to feel that the factor of mobility is being overemphasized, and the whole problem too much simplified.

Permit me once more to emphasize the changes that take place when a tooth is lost from the arch. There are changes in the opposing teeth, and in the occlusal plane, bringing it out of harmony with the planes of the remaining natural teeth; changes which are so slight as to be frequently overlooked when examining the mouth itself, and which should, therefore, receive special study when examining our models. When these changes are ignored, and I mean to include even the slightest distortion of the occlusal plane, as well as the dropping or elongation of an opposing tooth; and when the bridge is simply made so as to occlude when the teeth are in contact, then it is inevitable that the articulation should prove to be faulty. In that case, less harm will be caused to the abutment teeth, as well as to the opposing teeth, if both the abutments and the dummies be allowed individual movement—I had almost said, the more movement the better.

